# Neuroprotection afforded by targeting G protein-coupled receptors in heteromers and by heteromer-selective drugs

**DOI:** 10.3389/fphar.2023.1222158

**Published:** 2023-07-13

**Authors:** Rafael Franco, Gemma Navarro

**Affiliations:** ^1^ Molecular Neurobiology Laboratory, Department of Biochemistry and Molecular Biomedicine, Faculty of Biology, Universitat de Barcelona, Barcelona, Spain; ^2^ CiberNed, Network Center for Neurodegenerative Diseases, National Spanish Health Institute Carlos III, Madrid, Spain; ^3^ School of Chemistry, Universitat de Barcelona, Barcelona, Spain; ^4^ Department of Biochemistry and Physiology, School of Pharmacy and Food Science, Universitat de Barcelona, Barcelona, Spain; ^5^ Institute of Neurosciences, Universitat de Barcelona, Barcelona, Spain

**Keywords:** heteromer imprint, heteromer selective drug, GPCR, neuronal death, neurodegeneration, drug discovery, biased agonism

## Abstract

G protein-coupled receptors (GPCRs) are the target of hundreds of approved drugs. Although these drugs were designed to target individual receptors, it is becoming increasingly apparent that GPCRs interact with each other to form heteromers. Approved drug targets are often part of a GPCR heteromer, and therefore new drugs can be developed with heteromers in mind. This review presents several strategies to selectively target GPCRs in heteromeric contexts, namely, taking advantage of i) heteromer-mediated biased agonism/signalling, ii) discovery of drugs with higher affinity for the receptor if it is part of a heteromer (heteromer selective drugs), iii) allosteric compounds directed against the interacting transmembrane domains and, eventually, iv) antagonists that block both GPCRs in a heteromer. Heteromers provide unique allosteric sites that should help designing a new type of drug that by definition would be a heteromer selective drug. The review also provides examples of rhodopsin-like class A receptors in heteromers that could be targeted to neuroprotect and/or delay the progression of diseases such as Parkinson’s and Alzheimer’s. GPCRs in heteromers (GriH) with the potential to address dyskinesias, a common complication of dopaminergic replacement therapy in parkinsonian patients, are also described.

## Introduction

G protein-coupled receptors (GPCRs) are the target of 30%–40% of approved drugs. Early functionality studies related to neurotransmission/neuromodulation led to the hypothesis of receptor-receptor interactions (Agnati et al., 1982). Among the major GPCR families (Alexander et al., 2021), taste receptors form macromolecular complexes made up of at least two different receptors (Nelson et al., 2002). Class C GPCRs, such as metabotropic glutamate receptors do form dimers, both homo- and heterodimers. Dimer formation in class C GPCRs has been mainly addressed by studying the interactions established by large extracellular N-terminal domains. The first crystal structure of two interacting extracellular N-terminal domains of a class C receptor was reported in 2000 (Kunishima et al., 2000). Pharmacological studies performed in parallel to structural determination suggested that class C GPCR heteromerization “*is required to obtain a functional dimer as one of the subunits contains the endogenous ligand binding site, whilst the other mediates signal transduction heteromerization*” (Kniazeff et al., 2011). This view opened the question of whether GPCR/G protein stoichiometry was 1:1 or 2:1 (Cordomí et al., 2015).

With few exceptions, rhodopsin-like class A GPCRs do not have the huge N-terminal extracellular domains of class C GPCRs; hence, they form dimers through transmembrane and/or C-terminal domains (Stenkamp, 2018).

The first experimental evidence for class A heterodimer formation was provided for δ/κ opioid receptors (Jordan and Devi, 1999), and adenosine A_1_/dopamine D_1_ receptors (Gines et al., 2000). Heterodimerization of class A receptors has been questioned despite huge biochemical, pharmacological and biophysical evidence (Franco et al., 2016). The recent resolution of the structure of the homodimer of apelin receptor (Yue et al., 2022) has both confirmed that class A receptors may interact and that structure is important for signalling via the G proteins.

Adrenoceptors are the targets of several medicines. Are these medicines targeting monomeric adrenoceptors? It is reasonable to speculate that these medicines are targeting (cell surface) adrenoceptors that are expressed as monomers, as homodimers and as heteromers. For example, betaxolol is a selective antagonist of ß_1_ adrenoceptors, which can be expressed on the cell surface as homodimers or as complexes (heteromers) with ß_2_ adrenoceptors (Zhu et al., 2005).

A nice example of functional GPCR-containing macromolecular complex is the heterotetramer formed by two adenosine A_2A_ receptors and two adenosine A_1_ receptors coupled to one G_s_ and one G_i_ protein (Navarro et al., 2016; 2018b); the stoichiometry is two GPCRs:one G protein. There may be exceptions as deduced from the structure of a class D fungal receptor, Ste2, that reveals a homodimer coupled to two G proteins (Velazhahan et al., 2021). The structure and the stoichiometry of GPCR-G protein in oligomeric complexes is important for function (Yue et al., 2022). While it would be equivalent to target monomers or homomers, targeting heteromers makes possible to specifically target those cells that express them. On the one hand, targeting only cells expressing a given heteromer would reduce unwanted side effects and probably increase drug delivery options. On the other hand, targeting the GPCRs in heteromers (GRiH) of heteromers themselves can be achieved by quite different approaches, thus raising new possibilities for drug development.

## Properties derived from heteromerization. New perspectives for drug discovery strategies

Once a given heteromer is formed and appears on the cell surface, drug discovery must consider the properties of GriHs. The following properties should be considered: i) altered pharmacology, ii) cross-antagonism, iii) “altered” coupling to G proteins and iv) “altered” signaling.

At first it was assumed that the interaction of receptor A with receptor B could affect, even in the absence of a ligand of A, the ligand binding properties of B, or *vice versa*. There are allosteric modulations occurring when a heteromer is formed and, accordingly, the ligand binding affinity is, by definition, different between monomers/homodimers and heteromers (even for the same receptor in different heteromeric contexts). By the same token potency may be affected; the activation of a given pathway can even by blunted by heteromerization. After demonstrating that adenosine A_2A_ and A_3_ receptors do interact, we noticed that the G_i_-mediated signalling in response to A_3_ agonists does not occur unless the A_2A_ is blocked by an antagonists (Lillo et al., 2020).

The occurrence of heteromers in natural cells/environments can be identified by an imprint. The cross-antagonism imprint consists of blocking the function of a receptor by using an antagonist targeting the partner receptor in the heteromer. In other words, cross-antagonism for the AB heteromer, means that B function is blocked by the antagonist of A. The imprint occurring when cannabinoid CB_1_ and GPR55 interact consists of the blockade by a selective CB_1_ antagonist of the link of GPR55 to the mitogen-activated protein kinase signaling pathway. This imprint was observed in co-transfected cells and in striatal rat brain slices; further confirmation of the expression of the heteromer in striatal neurons was provided by an imaging technique using sections from *Macaca fascicularis* primates (Martínez-Pinilla et al., 2014).

Another possibility is differential G protein coupling. This property was described for dopamine D_1_-D_2_ receptor heteromers. While the D_1_ couples to G_s_ and the D_2_ couples to G_i_, the D_1_-D_2_ receptor heteromer couples to G_q_ and signals via Ca^2+^ instead of via cAMP (Rashid et al., 2007; Hasbi et al., 2009; Perreault et al., 2010; Perreault et al., 2015; Verma et al., 2010). Dopamine D_2_ and cannabinoid CB_1_ receptors when expressed individually couple to G_i_, but when they interact, the heteromer couples to G_s_ (Faron-Górecka et al., 2019). Co-activation of receptors leads to an increase in cytosolic cAMP levels, i.e., a G_s_-mediated effect; in cells where these heteromers are expressed, cannabinoids or D_2_ agonists increase rather than decrease cAMP levels (Caballero-Florán et al., 2016; Yu et al., 2021). Activation of a receptor in a heteromer may also affect regulation of ion fluxes and/or activation of the mitogen-activated protein kinase (MAPK) signaling pathway in a different way than if the receptor is expressed as a monomer/homodimer.

A type of functional selectivity known as biased signalling is also affected by the heteromeric context in which the GRiH is expressed. One of the advantages of targeting heteromers is that a given receptor may interact with several other receptors. An online tool allows finding the GPCR heteromers that have been reported to date (Borroto-Escuela et al., 2014). Accordingly, heteromeric-targeted drug discovery programs could take advantage of the possibility that there are compounds that have a higher affinity for binding to GRiH and/or that provide the most desirable signaling bias for therapeutic success. For instance, coactivation in a heteromer context of both cannabinoid CB_2_ and orexin OX_1_ receptors, for instance by dual drugs, results in a negative crosstalk at the level of the MAPK signaling pathway, favouring the G protein-dependent signalling (Raïch et al., 2022).

## GPCR heteromer-selective and bivalent molecules

**FIGURE 1:**
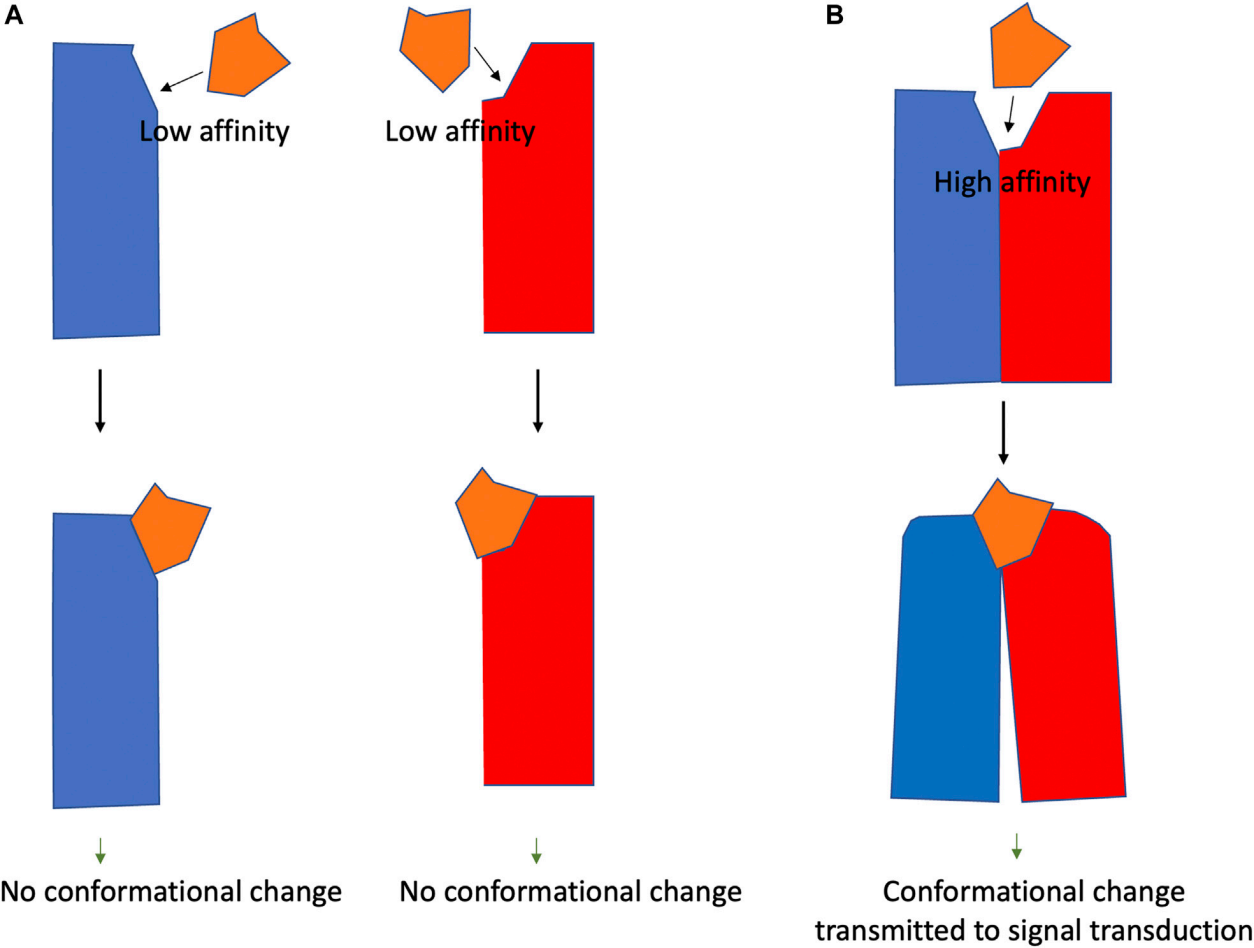
Heteromer-selective compounds. Panel **(A)**. The heteromer selective compound (orange) interacts with low affinity with the two GPCRs (blue and red). Panel **(B)**. The compound (orange) interacts with high affinity with the blue/red receptor heteromer and may lead to conformational changes.

Heteromer selective drugs are those that only interact with the structures arising from the interaction. As shown in Figure 1, a drug with a specific structure that cannot bind with high affinity to A or B receptors, could bind with high affinity to the AB heteromer. The design of selective heteromeric drugs with the potential to allosterically affect the binding of agonists to orthosteric centers or agonist potency is an attractive approach in future drug discovery.

Bivalent ligands that interact with two receptors in a heterodimer have been instrumental in detecting heteromers in the brain (Soriano et al., 2009; Kuhhorn et al., 2011). They are high-affinity probes with therapeutic potential, but they likely won't be developed to combat CNS diseases because chances of degradation before entering the brain. An alternative is the use of interfering peptides. A peptide containing both part of the sequence of an HIV-1 protein, transactivator of transcription (TAT), and the sequence of a GPCR transmembrane domain can be inserted into the plasma membrane. This type of peptide was already used in 2011 to demonstrate that disruption of the µ and ∂ opioid receptor heteromer affects analgesic effects of µ opioid receptor agonists (He et al., 2011). The question is whether these peptides able to modify the functionality of GRiH will be proposed and approved to enter clinical trials to assess efficacy in neurological diseases. A recent article provides clues on how to target transmembrane domains of GRiHs to obtain successful therapeutic drugs (Cai et al., 2023).

Considering the information presented above, altering the function of a given GRiH to meet a specific therapeutic requirement can be accomplished in at least four ways:

- Taking advantage of heteromer-mediated biased agonism/signalling (see previous section).
- Discovering heteromer selective drugs, i.e., drugs that only have high affinity for a GRiH.

There are few reported heteromer selective drugs and such paucity is mainly because selection is made for “monomers” rather than using heteromers. Early on, the laboratory of Susan George reported a selective agonist of a GRiH of the two forming the dopamine D_1_-D_2_ receptor heteromer (Verma et al., 2010). Subsequently, we reported A_2A_ receptor (A_2A_R) antagonists with differential binding/potency when tested on presynaptic A_2A_R heteromers in comparison with postsynaptic A_2A_R heteromers (Orru et al., 2011). Accordingly, it is suggested that drug screening be performed using the actual target, which in the case of GCPRs, would often be a GRiH.

- Altering the interaction interfaces in such a way that the GRiH function is altered to provide therapeutic benefits (see next section)
- Taking advantage of the cross-antagonism heteromer imprint (see next section)

## Heteromers with potential in neuroprotective approaches

A detailed description of all heteromers with potential in neurodegeneration is beyond the scope of this article; we have selected examples that illustrate both the therapeutic potential and the underlying mechanisms.

In what concerns hypoxia/ischemia/stroke, heteromers containing adenosine or serotonin receptors are involved in neuroprotective mechanisms. Preclinical experiments carried out years ago suggest the benefits of cannabidiol (CBD) in neonatal hypoxia (Castillo et al., 2010; Lafuente et al., 2011; Pazos et al., 2012). The mode of action is, at least in part, mediated by serotonin-HT_1A_/cannabinoid CB_2_ receptor heteromers, whose expression increases in a model of hypoxic-ischemic brain damage (Pazos et al., 2013; Franco et al., 2019). CBD is an allosteric modulator of the CB_2_ receptor that affects biased agonism depending on whether the receptor is expressed alone or it interacts with the most abundant cannabinoid receptor in the central nervous system (CNS), the CB_1_ (Martínez-Pinilla et al., 2017; Navarro et al., 2018c). In scenarios of increased expression of the CB_2_ receptor, for example, under neurodegenerative and/or ischemic conditions, CBD treatment attenuates brain damage (Campos et al., 2016). CBD also has beneficial effects on attenuating psychotic-, anxiety- and depressive behaviors. In summary, CBD itself or the derivatives recently reported (Navarro et al., 2021) have neuroprotective potential via cannabinoid GRiHs.

Drug addiction is one cause of neuronal cell death and attempts to minimize neurodegeneration have focused on GPCRs for neurotransmitters and neuromodulators expressed in reward circuits in the brain. Interestingly, *in vitro* disruption of a heteromer formed by two dopamine receptors, D_1_ and D_2_, promotes, increases, and accelerates locomotor activity, also enhancing the motivational effects induced by cocaine (Perreault et al., 2016). Consequently, a heteromer-selective agonist acting on dopamine GRiHs attenuates the action of the drug of abuse and the reinstatement of seeking behaviour (Hasbi et al., 2018). Thought to be segregated in different neuronal types, the D_1_ and the D_2_ receptors are coexpressed in a population of GABAergic neurons where they form heteromers (Rico et al., 2016); it is likely that the benefit on preventing cocaine noxious effects of activating dopamine receptors in the D_1_-D_2_ heteromer context is explained by key neuronal events that involve key proteins in dopaminergic neurotransmission, among others, extracellular signal-regulated kinase (ERK), ΔFosB and 32 kDa dopamine- and cAMP-regulated phosphoprotein (DARPP-32) (Hasbi et al., 2018). Another of the 5 existing dopamine receptor types, the D_3_, can form dimers with D_1_, D_2_ and other class A GPCRs. Apart from neuroprotection itself, D_3_ GRiHs are promising to manage dyskinesia, which is a common side effect of levodopa medication in Parkinson’s disease (PD) and that, at present, can only be treated by deep brain stimulation approaches (Scarselli et al., 2001; Fiorentini et al., 2003; Fiorentini et al., 2008b; Fiorentini et al., 2008a; Fiorentini et al., 2008c; Fiorentini et al., 2010; Fiorentini et al., 2013; Torvinen et al., 2005; Marcellino et al., 2008; Maggio et al., 2010; Maggio and Millan, 2010; Mutti et al., 2020).

Further examples of dopamine heteromers with potential in neuroprotection are those formed with adenosine receptors (ARs). These were among the first heteromers identified for receptors of two different endogenous agonists. In the basal ganglia neurons, the adenosine A_1_ interacts with the D_1_ receptor and the adenosine A_2A_R interacts with the D_2_ receptor. The therapeutic potential of targeting ARs in PD, disclosed several years ago, was based on the adenosine-dopamine antagonism at the CNS level. The lack of dopamine production in the disease would be partially reverted by using AR antagonists; AR blockade would potentiate the effect of dopamine produced by patients and/or the dopamine-replacement medication. After several attempts by different pharmaceutical companies and several clinical trials, a first in class AR antagonist, istradefylline, has been approved for human use (Jenner et al., 2009; Saki et al., 2013; Jenner, 2014; Mori et al., 2022) (marketed as Nouriast^®^ in Japan and as Nourianz^®^ in US; 20 mg tablets). Istradefylline targets the receptor in the striatum where it interacts with the dopamine D_2_ receptor and with other GPCRs. Istradefylline has been approved as adjuvant therapy in combination with levodopa, i.e., it has not been approved as a neuroprotective drug. Then the second relevant possibility is that this drug may have neuroprotective, i.e., disease-modifying, potential. On the one hand, the FDA does not have useful biomarkers for assaying neuroprotective potential. On the other hand, longitudinal studies that compare the progression of the disease in patients who take the drug versus those who do not (it is not approved in Europe), can confirm whether the neuroprotective potential shown by A_2A_R antagonists *in vitro* (Dall’lgna et al., 2003; Agnati et al., 2004; Armentero et al., 2011; Jenner, 2014) also occurs in patients with neurodegenerative diseases.

Much of the research aimed at providing neuroprotection in neurodegenerative diseases focuses on neurons even though microglia play a key role in neurodegenerative diseases, such as Parkinson’s (PD), Alzheimer’s (AD) and Huntington’s, which have a neuroinflammatory component. About two decades ago we reported that activation of the A_2A_R increases the nitric oxide release by activated primary microglia (Saura et al., 2005) and we discovered that the A_2A_R was upregulated in the microglia surrounding the plaques found in post-mortem brain samples from patients with AD (Angulo et al., 2003). We became interested since then in literature showing how pharmacological manipulation of A_2A_ would affect the proinflammatory/neuroprotective balance of microglia. Strong evidence comes from the detection of a limitation of the proinflammatory action of lipopolysaccharide after intracerebroventricular administration of a highly selective A_2A_R antagonist, SCH-58261 (Rebola et al., 2011). In a rodent model of PD, administration of A_2A_R antagonists reverses neuroinflammation accompanying 1-methyl-4-phenyl-1,2,3,6-tetrahydropyridine injury (Frau et al., 2011). Equivalent results, i.e., A_2A_R antagonists reversing the microglial inflammatory response, have been reported in another rodent model of striatal neurodegeneration (Minghetti et al., 2007). Based on these and other complementary results, whose description) is beyond the scope of this article, we recently noted that evidence for the neuroprotective potential of A_2A_R antagonists needs to be addressed both in microglia and in terms of targeting A_2A_R-containing heteromers. Another AR that is present in the microglia is the A_3_, which in the presence of the A_2A_ cannot signal through G proteins. These adenosine GRiHs expressed in the same cell are potential targets to prevent neuroinflammation**.** An interesting finding related to the A_2A_-A_3_ heteromer is that it is upregulated in primary microglia of the APP_Sw,Ind_ transgenic model of AD. The receptor heteromer functionality in primary microglia from the transgenic and from the control animals was similar (Lillo et al., 2022). Since the animal model does not present cognitive alterations until an advanced age, there must be protection that lasts for months until the system becomes unbalanced. Are microglia involved in brain circuit protection despite the expression of a noxious mutated amyloid precursor protein? Could activation or blockade of GRiHs in microglia protect neurons from severe impairment?

Another homotropic heteromer, formed by adenosine A_1_ and A_2A_ receptors seems to be involved in the *in vitro* neuroprotective potential of an endogenous nucleoside, guanosine (Massari et al., 2021). The *in vitro* benefits of guanosine, consists of reducing both mitochondrial alterations and oxidative stress. It would be relevant to know how guanosine exerts its effects to address the possibility of design novel ligands targeting adenosine GRiH. It is relevant to i) select the right heteromer and the right GRiH(s) to be targeted to obtain a given therapeutic outcome and ii) be sure that the heteromer is expressed in the cells that must be targeted in the intended therapeutic benefit.

Cannabinoid CB_1_ and CB_2_ receptors forming CB_1_-CB_2_ heteromers in microglia have potential as targets for AD therapy. They are upregulated in both activated microglia and in the microglia isolated from the hippocampus from a rodent model of the disease (Navarro et al., 2018a). Other receptors that are targeted by phytocannabinoids although they are not considered as cannabinoid receptors, GPR18 and GPR55, can form heteromers with cannabinoid receptors. Their expression in brain cells and in animal models of neurodegenerative diseases indicate that those GRiH must be considered in disease-modifying neuroprotective approaches to combat PD and/or AD (Reyes-Resina et al., 2018; Martínez-Pinilla et al., 2019; Pérez-Olives et al., 2021).

## Heteromers of potential relevance, awaiting further confirmation

The bradykinin B_2_ receptor and the κ opioid receptor, which are co-expressed in several brain regions, can form heteromers whose potential as a neuroprotective target stems from the fact that the major endogenous κ-opioid receptor agonist, dynorphin, is involved in the regulation of neuronal fate (Ji et al., 2017). Finally, the 2 types of angiotensin II receptors, AT_1_ and AT_2_, are expressed in various brain regions and are of special interest in relation to the pathophysiology of Parkinson’s disease. The two receptors can form heteromers that are upregulated in the unilateral 6-hydroxydopamine lesioned Parkinson’s disease model. Boosting the neuroprotective potential of AT_2_ receptors may be more successful when heteromers and GRiHs are considered (Rivas-Santisteban et al., 2020).

## Abbreviations

AR, Adenosine receptor; CBD, Cannabidiol; CNS, central nervous system; GPCR, G protein-coupled receptor; GRiH, GPCRs in heteromers; TAT, transactivator of transcription (TAT); TM, transmembrane domain.
